# Enhanced design of multiplexed coded masks for Fresnel incoherent correlation holography

**DOI:** 10.1038/s41598-023-34492-2

**Published:** 2023-05-06

**Authors:** Shivasubramanian Gopinath, Andrei Bleahu, Tauno Kahro, Aravind Simon John Francis Rajeswary, Ravi Kumar, Kaupo Kukli, Aile Tamm, Joseph Rosen, Vijayakumar Anand

**Affiliations:** 1 Institute of Physics, University of Tartu, W. Ostwaldi 1, 50411 Tartu, Estonia; 2 Department of Physics, SRM University-AP, Amaravati, Andhra Pradesh 522502 India; 3 School of Electrical and Computer Engineering, Ben-Gurion University of the Negev, 8410501 Beer-Sheva, Israel; 4 Optical Sciences Center and ARC Training Centre in Surface Engineering for Advanced Materials (SEAM), School of Science, Computing and Engineering Technologies, Optical Sciences Center, Swinburne University of Technology, Hawthorn, Melbourne, VIC 3122 Australia

**Keywords:** Optical techniques, Imaging and sensing, Lithography, Microscopy

## Abstract

Fresnel incoherent correlation holography (FINCH) is a well-established incoherent digital holography technique. In FINCH, light from an object point splits into two, differently modulated using two diffractive lenses with different focal distances and interfered to form a self-interference hologram. The hologram numerically back propagates to reconstruct the image of the object at different depths. FINCH, in the inline configuration, requires at least three camera shots with different phase shifts between the two interfering beams followed by superposition to obtain a complex hologram that can be used to reconstruct an object’s image without the twin image and bias terms. In general, FINCH is implemented using an active device, such as a spatial light modulator, to display the diffractive lenses. The first version of FINCH used a phase mask generated by random multiplexing of two diffractive lenses, which resulted in high reconstruction noise. Therefore, a polarization multiplexing method was later developed to suppress the reconstruction noise at the expense of some power loss. In this study, a novel computational algorithm based on the Gerchberg-Saxton algorithm (GSA) called transport of amplitude into phase (TAP-GSA) was developed for FINCH to design multiplexed phase masks with high light throughput and low reconstruction noise. The simulation and optical experiments demonstrate a power efficiency improvement of ~ 150 and ~ 200% in the new method in comparison to random multiplexing and polarization multiplexing, respectively. The SNR of the proposed method is better than that of random multiplexing in all tested cases but lower than that of the polarization multiplexing method.

## Introduction

Multifunctional diffractive and holographic optical elements play a vital role in imaging, holography, beam shaping, optical trapping, augmented reality (AR) and virtual reality (VR) applications^1–10^. The combination of different diffractive phase masks to achieve multifunctionality is often a challenging task during implementation. One of the well-established methods for combining two diffractive functions is the modulo-2π phase addition method given as ${\Phi }_{T}={\left[{\Phi }_{1}+{\Phi }_{2}\right]}_{2\pi }$. The proposed approach is suitable for combining two greyscale diffractive functions^3,5,10^, a greyscale diffractive function with a binary diffractive function^7^ and two binary diffractive functions^3^. In all the above cases, the resulting diffractive function can be implemented as a greyscale diffractive function and binary diffractive function with corresponding maximum diffraction efficiencies of 1 and 0.4, respectively^11^. The above modulo-2π phase addition method transfers the far-field intensity and phase distribution of the diffraction patterns of one function to the other. For instance, in microdrilling applications, an array of ring patterns is created by combining a Damman grating with a diffractive axicon, and the dot patterns generated by the Dammann grating are replaced by the ring patterns of the axicon^12^. The diffraction effect of one element is intertwined with that of the other, which results in surprising behaviors in certain cases, such as quasi-achromatic focusing of light^3,13^.

It is often desirable for certain applications, such as incoherent holography, optical communication, and cryptography, to combine two diffractive functions such that they behave independently of one another. One direct approach to combine two diffractive functions with independent behavior is to directly sum the two pure phase functions as $\psi =\mathrm{exp}(i{\Phi }_{1})+\mathrm{exp}(i{\Phi }_{2})$. However, the resulting function is not phase-only but complex with variations in both magnitude and phase. In principle, such a modulation is possible to realize, but requiring both amplitude and phase modulation makes the implementation challenging, as both active optical modulators such as spatial light modulators (SLMs) are either phase-only or amplitude-only. Even with micro/nanofabrication methods, realizing such a complex function requires a minimum of two optical elements with precalculated spacing between them. The efficiency of such a configuration is expected to be lower than that of single element configurations. Alternative methods were developed to overcome this challenge. One widely used method to combine two diffractive functions such that they exhibit independent behavior is random multiplexing^14,15^. In the random multiplexing method, a random mask is generated, and different diffractive functions are encoded to pixels of the random mask with a certain range of values. By selecting the ratio between the number of pixels assigned to different diffractive functions, it is possible to effectively control the optical power splitting across the diffractive functions.

In^14^, randomly multiplexed diffractive lenses were used to demonstrate a motionless, nonscanning incoherent digital holography technique called Fresnel incoherent correlation holography (FINCH). The light from an object was modulated by the two diffractive lenses independent of one another, resulting in two different object waves derived from the same object point, which interfered to form a self-interference hologram. In^15^, the same technique was implemented using randomly multiplexed diffractive lenses manufactured using the electron-beam lithography technique as supposed to be in an SLM^14^. Both cases^14^ and^15^ suffered from disturbing background noise during recording of the hologram as well as its reconstruction. Recently, a random multiplexing approach has been used to increase the speed of singular beam generation^16^. The random multiplexing approach has multiple advantages, such as the capability to control the power splitting between multiple multiplexed functions, pure phase multifunctional diffractive functions and easy implementation. However, the noise generated due to random multiplexing and the associated power loss are not desirable for many imaging, holography, beamshaping, and AR/VR applications. Consequently, a different approach called the polarization multiplexing method was developed to remove the background noise in FINCH at the expense of lower light efficiency^17,18^. In the polarization multiplexing method, two polarizers are used: one to polarize the incoming unpolarized light from the light source at 45° with respect to the active axis of the SLM and another just before the image sensor to create interference between modulated and unmodulated light from the SLM. The use of two polarizers reduces the intensity by approximately 50% at every pass, resulting in a light throughput of only 25%. The random multiplexing method, on the other hand, uses a single polarizer and thus has a light throughput of 50%. In addition, polarization multiplexing is often limited to two diffractive functions, whereas random multiplexing can be used for more than two diffractive functions.

In this study, a novel computational algorithm based on the Gerchberg-Saxton algorithm (GSA)^19^ called transport of amplitude into phase using GSA (TAP-GSA) has been developed to multiplex multiple diffractive functions. The proposed algorithm begins with the complex function *ψ* obtained by a summation of different diffractive phase-only functions and iteratively encodes the amplitude information into the phase information with amplitude and phase constraints in the sensor plane. The method has been implemented on FINCH but can also benefit other incoherent holography techniques, such as interferenceless coded aperture correlation holography (I-COACH)^20^ with dot patterns^21^ and Airy beams^22^. The manuscript consists of “Conclusion and future perspectives” sections. The methodology is described in the next section. The simulation studies for FINCH are presented in the “Simulation studies” section. In the fourth section, optical experiments are discussed, and the experimental results are presented. The conclusion and future perspectives of the study are presented in the final section.

## Methodology

**Figure 1:**
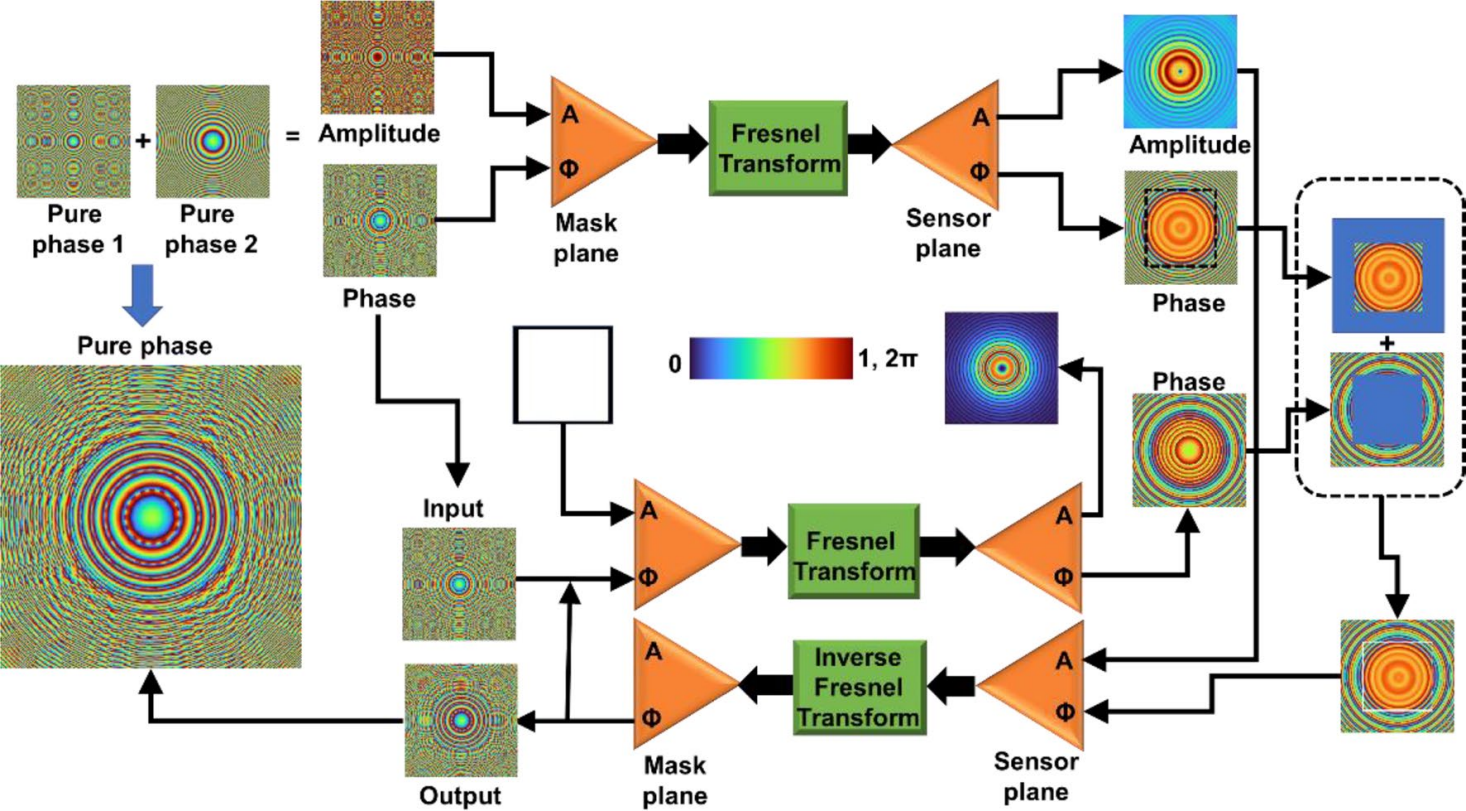
Schematic of TAP-GSA. Two pure phase functions are added to create a complex function. The phase of the complex function and a uniform matrix were used as phase and amplitude constraints, respectively, in the mask domain. The amplitude distribution obtained by Fresnel propagation of the ideal complex function to the sensor domain is used as a constraint in the sensor domain. The phase distribution obtained at the sensor plane by Fresnel propagation is combined with the ideal phase distribution.

The schematic of TAP-GSA is shown in Fig. 1. The algorithm consists of two steps. In the first step, two or more pure phase functions are summed, resulting in a complex function, as shown at the top of Fig. 1. This complex function is the ideal function in the mask plane. In the shown case, two pure phase functions named ‘pure phase 1’ and ‘pure phase 2’ are used. The resulting complex function is numerically propagated from the mask plane using a Fresnel propagator to a distance, as required in an optical experiment, to the sensor plane, and the resulting complex amplitude is the ideal function at the sensor plane. The TAP-GSA begins at the mask plane with a uniform amplitude and a phase extracted from the phase of the ideal complex function. The resulting complex amplitude at the mask plane is propagated to the sensor plane using a Fresnel propagator, and the resulting amplitude is replaced by the ideal amplitude calculated at the sensor plane for the ideal complex function at the mask plane. The resulting phase after Fresnel propagation is combined with the ideal phase, as shown in Fig. 1. The key differences between GSA and TAP-GSA are the following: (a) Mostly GSA is used with the Fourier transform operation between the two planes of interest, while TAP-GSA uses the Fresnel transform operation between the two planes of interest. (b) In most GSA applications, the initial guess is a random phase function, whereas in TAP-GSA, the initial guess is the phase of the ideal complex function. This approach reduces randomness in the mask. (c) In most applications, GSA is used to achieve a certain intensity distribution with almost no constraint on the phase in the sensor plane, whereas since the current application is holography, the phase information is crucial to reconstruct images at different planes. Therefore, a limited phase constraint is also placed at the sensor plane in TAP-GSA. (d) As the name suggests, TAP-GSA has a specific application, which is to transport the amplitude information into phase, creating a phase-only function from a complex function, while most applications of GSA focus on calculating the phase distribution at the mask plane to obtain a certain intensity distribution at the sensor plane.

**Figure 2:**
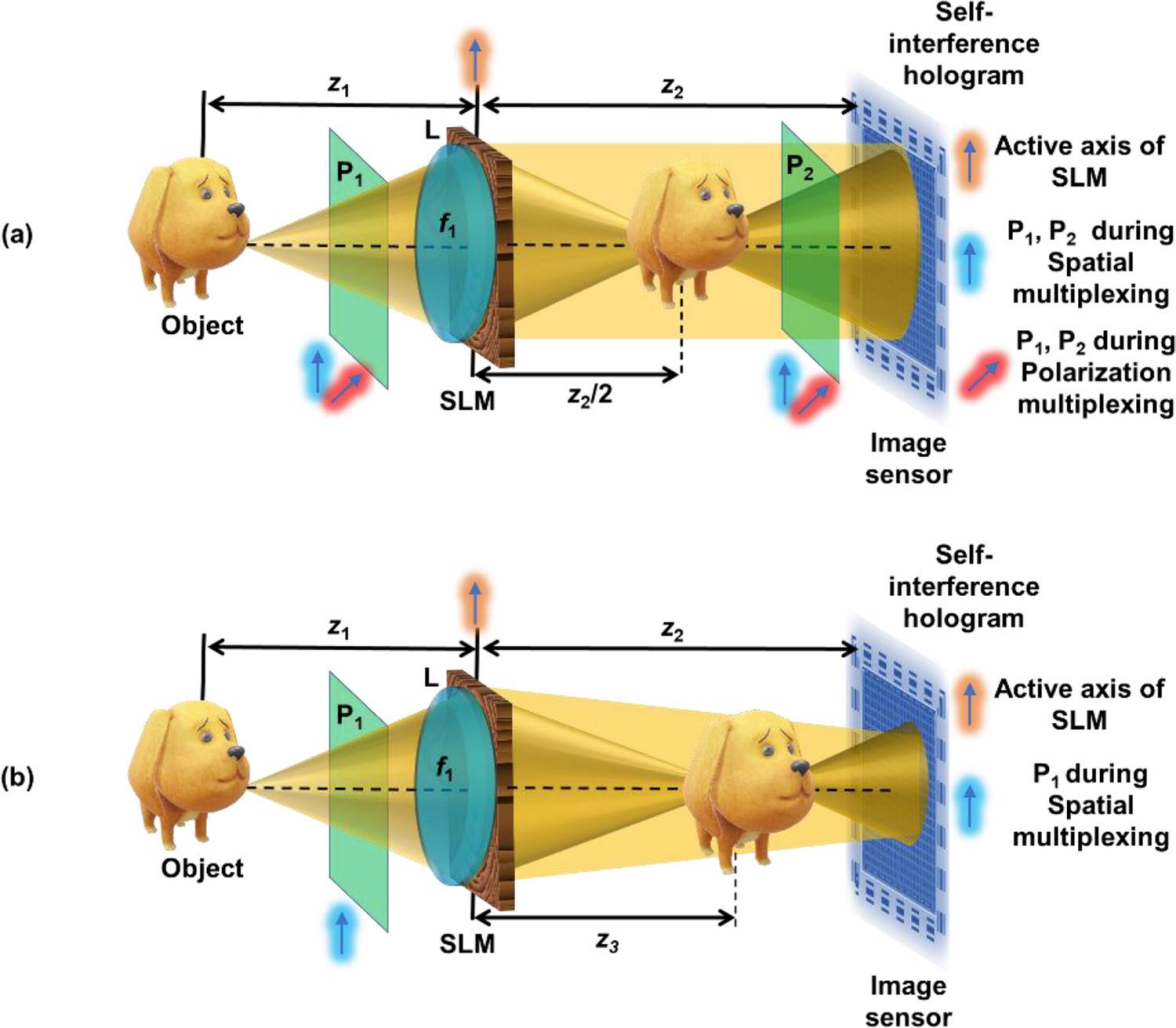
Optical configurations of FINCH with (**a**) maximum path difference and (**b**) reduced path difference.

Two different optical configurations of FINCH are considered in this case, namely, FINCH with maximum path difference^17^ and FINCH with reduced path difference^23^, as shown in Fig. 2a and b, respectively. The two configurations of FINCH are indirect imaging systems in the sense that the image is reconstructed by a computer algorithm in contrast to a direct imaging system in which the image is directly obtained on the sensor plane. In FINCH, light from an object point is split into two beams, and each beam is focused using a diffractive lens with a different focal length than the other beam. The resulting two differently modulated object waves interfere, creating the self-interference hologram. For FINCH, in the in-line configuration, at least three camera recordings with phase shifts (*θ* = 0, 2π/3 and 4π/3) are needed, which are superposed to generate a complex hologram. This complex hologram numerically propagates to different distances to reconstruct the object images at different planes^17^.

For the first configuration (Fig. 2a), a point object with an amplitude of $\sqrt{{I}_{o}}$ is considered. The complex amplitude of the point object at a distance of *z*_1_ from the plane of a refractive lens having a focal length *f*_1_ is given as ${C}_{1}\sqrt{{I}_{o}}Q\left(1/{z}_{1}\right)$, where $Q\left(a\right)=\mathrm{exp}\left(i\pi {ar}^{2}/\lambda \right)$, $r=\sqrt{{x}^{2}+{y}^{2}}$ and *C*_1_ is a complex constant. For the polarization multiplexing method, the modulation function can be expressed as $\mathrm{exp}\left(i\pi {r}^{2}/\lambda {f}_{2}\right)$ $\mathrm{for}$ one orientation and constant for the other orthogonal orientation. The phase function displayed on the SLM for the random multiplexing method is given by $\Phi (x,y)=B(x,y)+\left(\pi {r}^{2}/\lambda {f}_{2}\right)\left(1-B(x,y)\right)$, where $B(x,y)\in \left\{\mathrm{0,1}\right\}$ is a binary random function. The first term of $\Phi (x,y)$ does not change the incoming light, while the second term focuses light at z_2_/2 when* f*_2_ satisfies the relation (*f*_1_ + *f*_2_)/(*f*_1_*f*_2_) = 1/*z*_1_ + 2/*z*_2_, and in the case *z*_1_ = *f*_1_, *f*_2_ = *z*_2_/2. The complex amplitude after the SLM is given as ${C}_{2}\sqrt{{I}_{o}}Q\left(1/{z}_{1}-1/{f}_{1}\right)\mathrm{exp}\left[-i\Phi (x,y)\right]$, where *C*_2_ is a complex constant. The point spread function is given as $I_{{{\text{PSF}}}} = \left| {C_{2} \sqrt {I_{o} } Q\left( {1/z_{1} - 1/f_{1} } \right){\text{exp}}\left[ { - i\Phi \left( {x,y} \right)} \right] \otimes Q\left( {1/z_{2} } \right)} \right|^{2}$, where ‘$\otimes$’ is a 2D convolutional operator. As FINCH is a linear shift-invariant system, the hologram *H* for an object *O* can be given as $I_{{{\text{PSF}}}} \otimes O$. Since FINCH uses an in-line configuration, at least three holograms *H*_1_, *H*_2_ and *H*_3_ with phase shifts *θ* = 0, 2π/3 and 4π/3 are recorded and combined as $H_{C} = H_{1} \left( {{\text{exp}}\left[ { - i4\pi /3} \right] - {\text{exp}}\left[ { - i2\pi /3} \right]} \right) + H_{2} \left( {1 - {\text{exp}}\left[ { - i4\pi /3} \right]} \right) + H_{3} \left( {{\text{exp}}\left[ { - i2\pi /3} \right] - 1} \right)$ to obtain a complex hologram. The image of the object is reconstructed from the hologram as $I_{R} = H_{C} \otimes Q\left( {1/z_{R} } \right),$ where *z*_*R*_ is the reconstruction distance. By tuning the reconstruction distance, it is possible to reconstruct different planes of the object. For the second configuration (Fig. 2b), with the polarization multiplexing method, the modulation function can be expressed as $\mathrm{exp}\left(i\pi {r}^{2}/\lambda {f}_{2}\right) \mathrm{for}$ one orientation and $\mathrm{exp}\left(i\pi {r}^{2}/\lambda {f}_{3}\right)$ for the other orthogonal orientation. For the random multiplexing method and assuming* z*_1_ = *f*_1_, the phase function displayed on the SLM is given as $\Phi (x,y)=\left(\pi {r}^{2}/\lambda {f}_{2}\right)B(x,y)+\left(\pi {r}^{2}/\lambda {f}_{3}\right)\left(1-B(x,y)\right)$, where ${f}_{2}^{-1}+{f}_{3}^{-1}=2{z}_{2}^{-1}$ to satisfy the beam matching condition^23^. The first image is formed at a distance *z*_3_ (= *f*_2_ in case *z*_1_ = *f*_1_) from the SLM. The above two configurations are used to compare the performances of the random multiplexing method with the proposed new method using TAP-GSA. For the first configuration, three cases, namely, polarization multiplexing, random multiplexing and the new approach using TAP-GSA, were compared. For the second configuration, random multiplexing and the new method are compared.

## Simulation studies

A detailed simulation study of the TAP-GSA was carried out using MATLAB^24^. A matrix consisting of 500 × 500 pixels was used with a pixel size of 10 μm and a wavelength λ = 632.8 nm. In the simulation, there is no difference between the first and second configurations, as a single wavelength has been used. The optical configuration was simulated for distances of *z*_1_ = 30 cm, *z*_2_ = 60 cm and *f*_1_ = ∞, *f*_2_ = 30 cm and *f*_3_ = 15 cm. The diameter of the entrance pupil was 2.5 mm. Different cases were considered for the simulation study, such as random multiplexing, polarization multiplexing, and spatial multiplexing using TAP-GSA with different degrees of freedom (DOFs). The DOF is defined as the ratio between the number of pixels replaced in the phase matrix of the sensor and the total number of pixels in the matrix. The DOF was varied as 19, 36, 51, 84, and 100% with the initial guess of the ideal phase and 100% with the initial guess as a random matrix. In random multiplexing, diffractive lenses with focal distances *f*_2_ and *f*_3_ are multiplied to a binary random matrix [0,1] and its inversion matrix [1,0], respectively, and summed to form the phase matrix. In polarization multiplexing, the ideal complex function formed by a summation of the phases of the two diffractive lenses was used as the modulation function. In TAP-GSA-based spatial multiplexing, a uniform amplitude constraint is placed in the mask plane, and the amplitude obtained at the sensor plane for polarization multiplexing is used as a constraint on the sensor plane. The DOF of the phase constraint in the sensor plane was varied from 100 to 19%, and the phase mask was calculated after 100 iterations.

**Figure 3:**
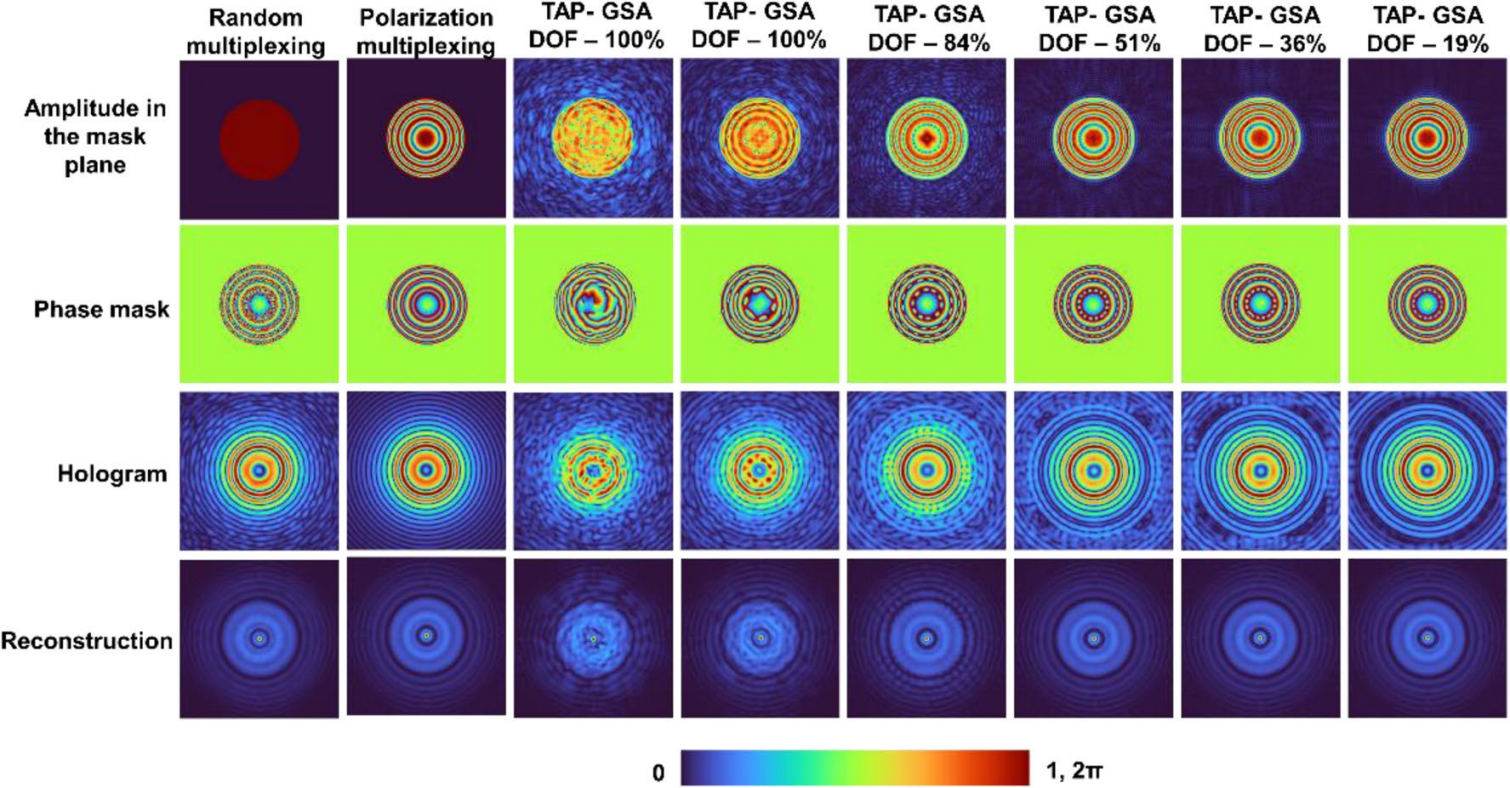
Simulation results of random multiplexing, polarization multiplexing and spatial multiplexing using TAP-GSA with different DOFs of 100, 84, 51, 36 and 19%.

**Figure 4:**
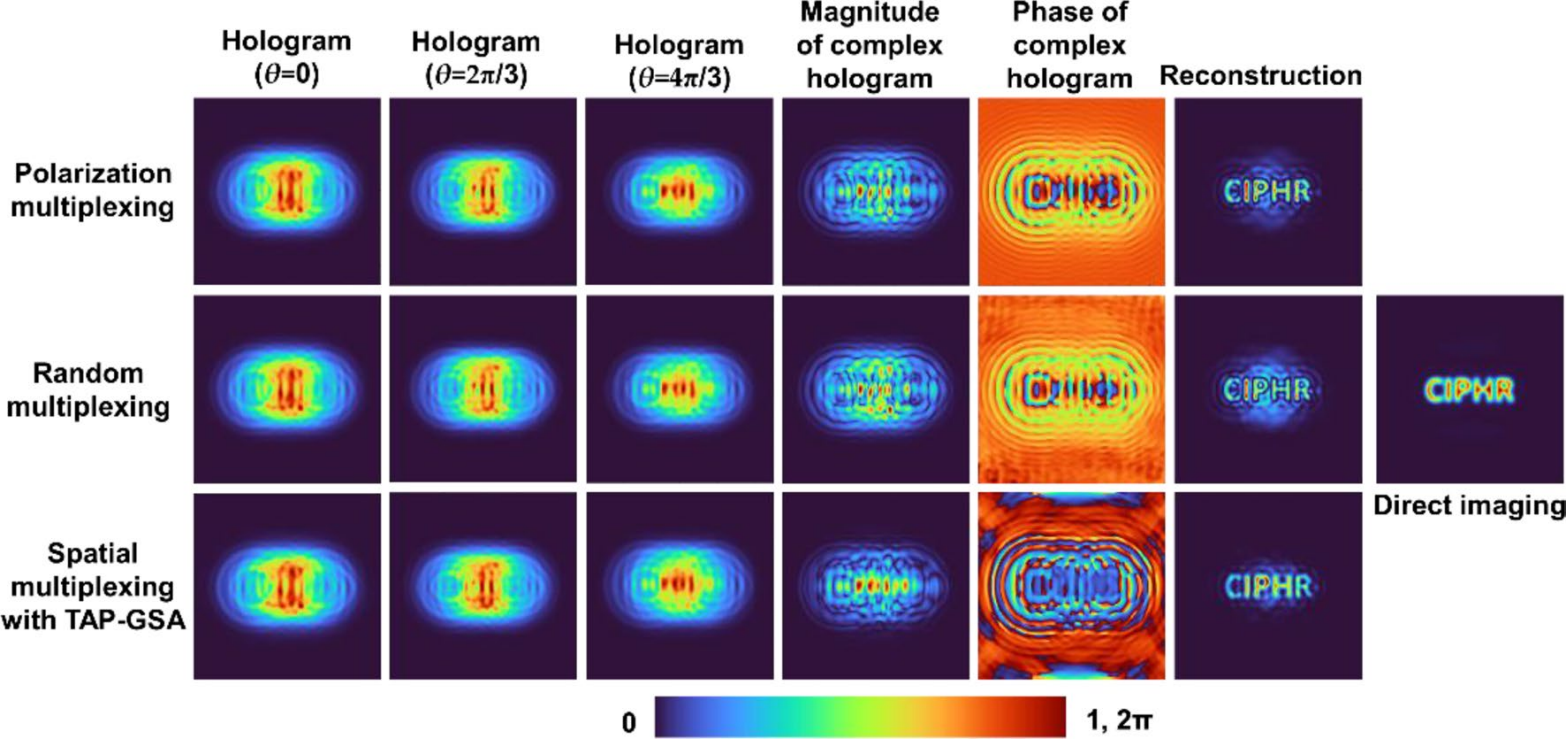
Simulation results of holograms with phase shifts *θ* = 0, 2π/3 and 4π/3, the magnitude and phase of the complex hologram and the reconstruction results by Fresnel back propagation for polarization multiplexing, random multiplexing and spatial multiplexing with TAP-GSA.

As shown in Fig. 3, with an increase in the DOF, the amplitude at the mask plane became more uniform, but at the same time, the hologram became noisier. Consequently, the reconstructions for the cases with a high DOF are noisier than the other cases. Another important observation made from Fig. 3 is that the cases with minimum DOF, even though they do not have a uniform amplitude at the mask plane, generate a hologram identical to the ideal case. This is an interesting observation, as the implementation of the minimum DOF case does not require polarization multiplexing but has a high SNR and improves the light throughput by 200%. The simulation was extended to a test object with the letters ‘CIPHR’. The simulated holograms (*θ* = 0, 2π/3 and 4π/3) for polarization multiplexing, random multiplexing and spatial multiplexing with TAP-GSA with a DOF of 10%, the magnitude and phase of the complex hologram and the reconstructed image using Fresnel back propagation are shown in Fig. 4. As seen from the reconstruction results, the proposed method with spatial multiplexing with TAP-GSA has less reconstruction noise than random multiplexing, but the sharpness is slightly lower than that of polarization multiplexing. The resolution of all the cases is better than that of direct imaging, as expected, as FINCH has a 1.5 times higher resolution than the direct imaging system with the same numerical aperture.

## Experiments

**Figure 5:**
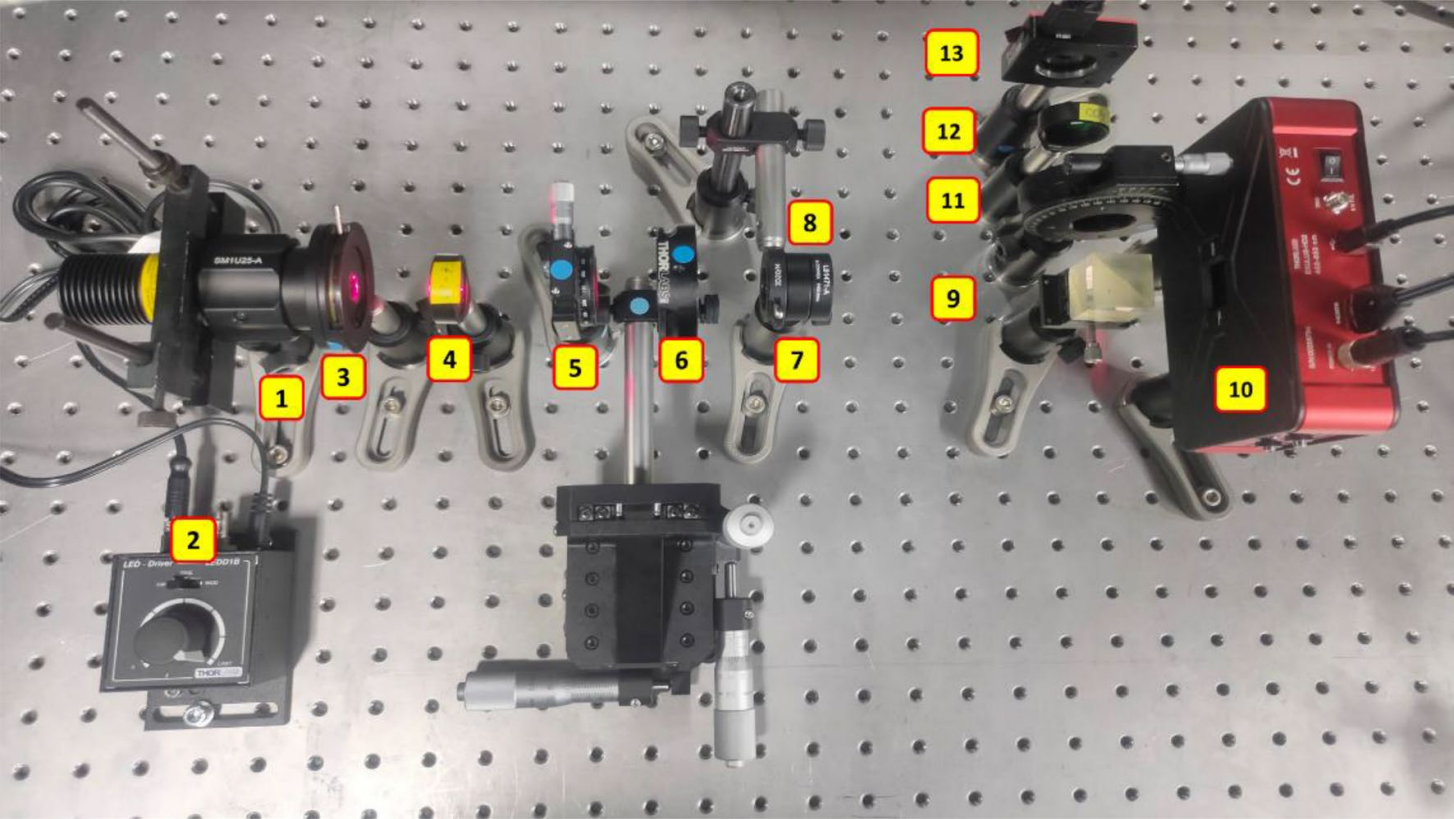
Photograph of the experimental setup: (1) LED, (2) LED power controller, (3) iris, (4) refractive lens (*f* = 50 mm), (5) polarizer P1, (6) object/pinhole, (7) iris, (8) refractive lens (*f* = 50 mm), (9) beam splitter, (10) SLM, (11) polarizer P2, (12) bandpass filter, and (13) image sensor.

The FINCH experimental setup was built on an optical table, and its photograph is shown in Fig. 5. The setup uses a high-power collimated red LED (Element 1) (Thorlabs, 170 mW, *λ* = 650 nm and Δ*λ* = 20 nm), which critically illuminates the object (Element 6) using a refractive biconvex lens (Element 4) with a focal length of 5 cm. The optical power controller shown is Element 2. Two objects, namely, a pinhole with *ϕ* = 100 μm (Thorlabs) and USAF object (Group–5, Element 1) number 5 and gratings with a line width of 15.63 μm, are used. An iris was used after the LED to control the illumination diameter (Element 3). A polarizer (P1) (Element 5) was used to allow light with a certain polarization orientation into the system. The polarizer P1 is oriented along the active axis of the SLM (Element 10) (Thorlabs Exulus HD2, 1920 × 1200 pixels, pixel size = 8 μm) for random multiplexing and spatial multiplexing with TAP-GSA and at 45° with respect to the active axis of the SLM for polarization multiplexing methods. The light from the object is collected by a biconvex refractive lens (Element 8) with a focal length of 5 cm located at a distance of 5 cm from the object. An iris (Element 7) was used in tandem with the lens to control the numerical aperture of the system. The light from the biconvex refractive lens is incident on a beamsplitter (Element 9) located at a distance of 13 cm and then the SLM, which is 5 cm from the beamsplitter. The light modulated by the SLM and redirected by the SLM is incident on the image sensor (Zelux CS165MU/M 1.6 MP monochrome CMOS camera, 1440 × 1080 pixels with pixel size ~ 3.5 µm) (Element 13) at a distance of 12 cm from the beamsplitter. The second polarizer P2 (Element 11) is used at 45° with respect to the active axis only for the polarization multiplexing method and removed in the cases of random multiplexing and spatial multiplexing with TAP-GSA. A bandpass filter (λ_c_ = 632.8 nm and Δλ = 5 nm) (Element 12) was used to improve the temporal coherence and thus the fringe visibility of the hologram. The phase masks were synthesized in the computer for both configurations of FINCH with reduced and maximum path differences. For reduced path difference, only random multiplexing and spatial multiplexing with TAP-GSA were compared. For maximum path difference, random multiplexing, spatial multiplexing with TAP-GSA and polarization multiplexing were compared. For the main experiment, the LED current was set to 0.2 A and the entire dynamic range of 1024 levels of the image sensor was used for recording holograms and direct images. To have a reliable comparison of exposure times for all measurements, the current of the LED driver was increased to 0.5 A, and the signal level (256 levels) in the image sensor was maintained at the same level to achieve the same baseline for all measurements.

### FINCH with reduced path difference

**Figure 6:**
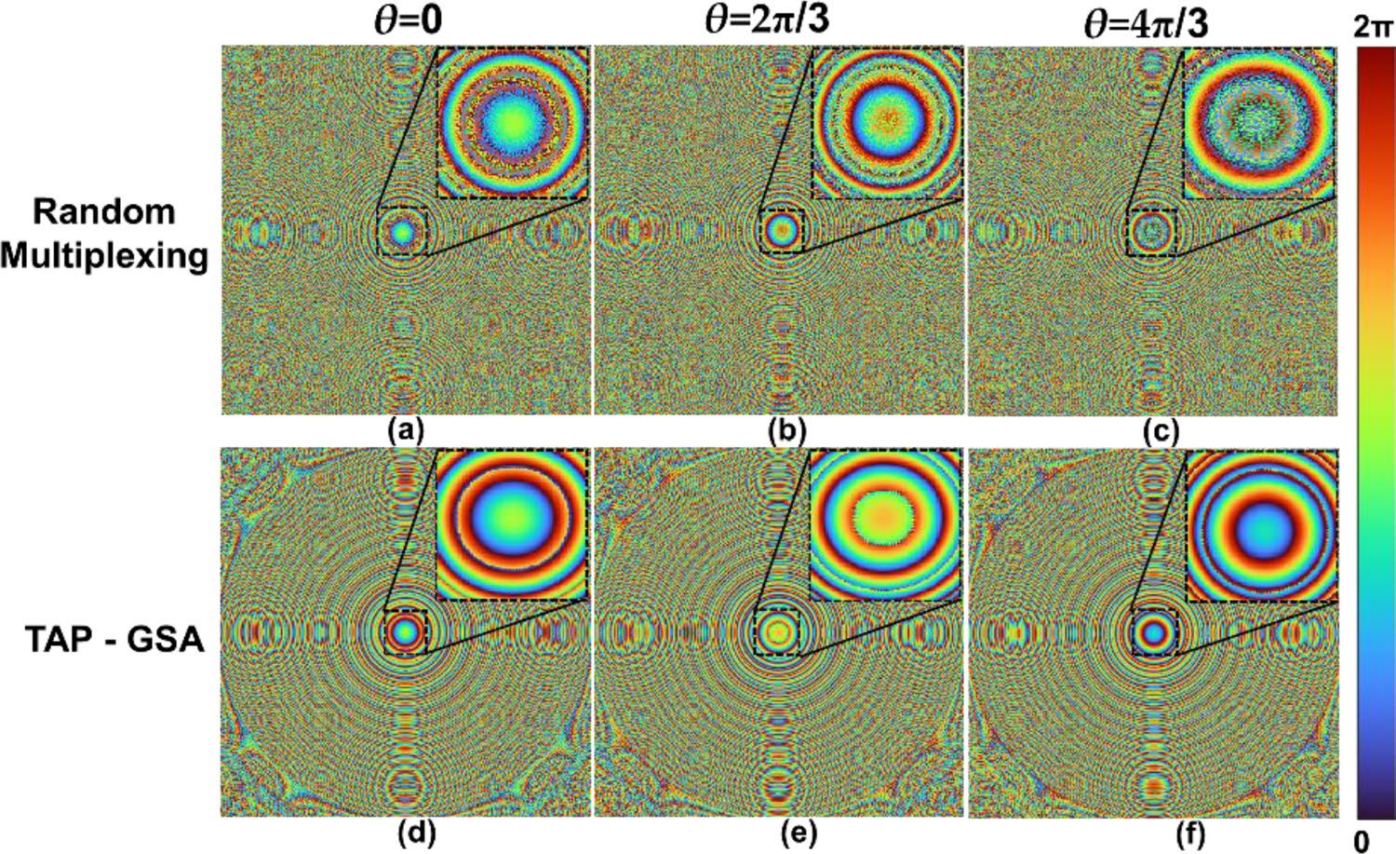
Phase masks for the setup of the reduced path difference. Two diffractive lenses with focal lengths of 14 and 25 cm are multiplexed. The upper line shows the phase masks designed using the random multiplexing method with phase shifts (**a**) *θ* = 0, (**b**) 2π/3 and (**c**) 4π/3. The lower line shows the phase masks designed using the TAP-GSA method with phase shifts of (**d**) *θ* = 0, (**e**) 2π/3 and (**f**) 4π/3.

The distance between the SLM and the image sensor and the nature of the incoming light to the SLM were analysed by displaying diffractive lenses with different focal lengths on the SLM. A diffractive lens with a focal length of 17.8 cm generated the best focus direct images. For the reduced path difference FINCH case, two diffractive lenses were designed with focal lengths of 14 and 25 cm. The two lenses were combined using random multiplexing and spatial multiplexing with TAP-GSA. Phase-shifted phase masks were synthesized by phase shifting the lens of 14 cm with *θ* = 0, 2π/3 and 4π/3. The phase images of the masks designed for random multiplexing for *θ* = 0, 2π/3 and 4π/3 are shown in Fig. 6a–c, respectively. The phase images of the masks designed for spatial multiplexing with TAP-GSA (DOF ~ 30%) for *θ* = 0, 2π/3 and 4π/3 are shown in Fig. 6d–f, respectively.

**Figure 7:**
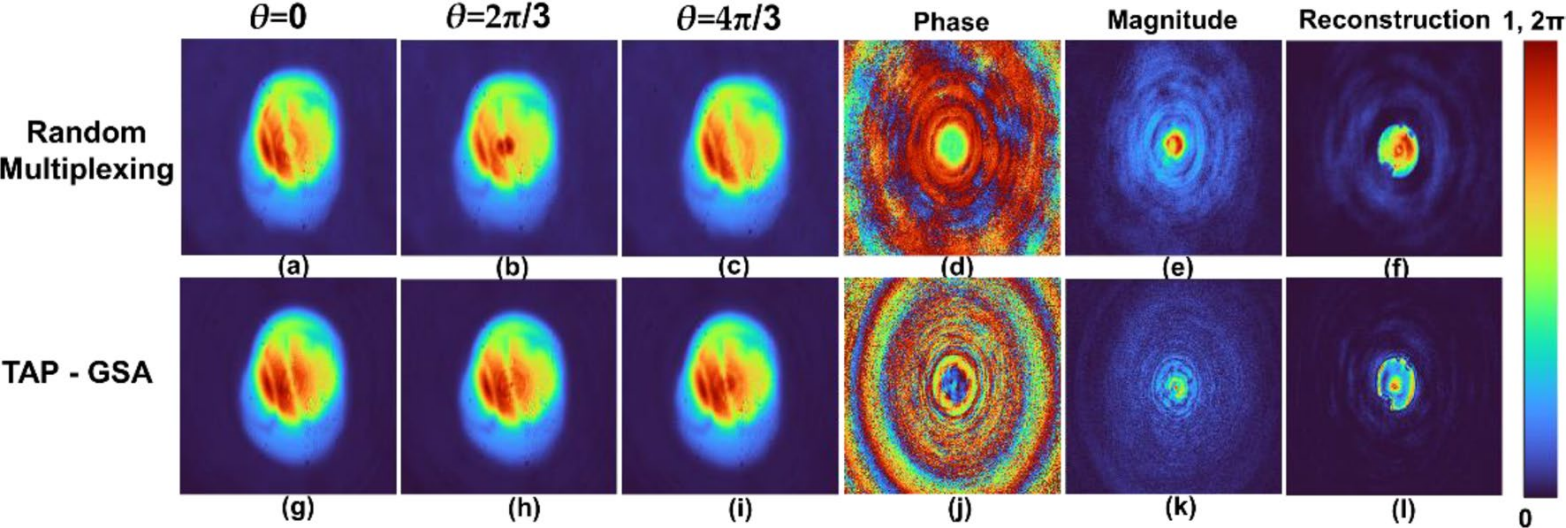
Holograms of the pinhole recorded with reduced path difference and the masks of Fig. 6. The upper line shows the holograms of the random multiplexing method with phase shifts (**a**) *θ* = 0, (**b**) 2π/3 and (**c**) 4π/3. (**d**) Phase and (**e**) magnitude of the complex hologram and (**f**) reconstructed image of the pinhole. Holograms of a pinhole by the TAP-GSA method with phase shifts (**g**) *θ* = 0, (**h**) 2π/3 and (**i**) 4π/3, (**j**) phase, (**k**) magnitude, and (**l**) reconstructed image of pinhole.

A pinhole object was mounted, and FINCH holograms with different phase shifts were recorded one after another. The recording and reconstruction results for the pinhole are shown in Fig. 7. For random multiplexing, the phase-shifted holograms of the pinhole with *θ* = 0, 2π/3 and 4π/3 are shown in Fig. 7a–c. The phase and magnitude of the complex hologram and the reconstruction result by Fresnel back propagation are shown in Fig. 7d–f, respectively. The same images as Fig. 7a–f for the method of spatial multiplexing with the TAP-GSA are shown in Fig. 7g–l. The reconstruction distance was approximately 7 cm. The average background noise (ABN) was estimated using the equation $ABN=\left\{\sum_{i=1,j=1}^{N,M}{I}_{R}\left({x}_{i},{y}_{j}\right)\right\}/(N\times M)$, where ${I}_{R}\left({x}_{i},{y}_{j}\right)$ is the value of pixel (*i,j*) if and only if pixel (*i,j*) is in the background of the image. In the case of the pinhole, ABNs are 2.36 × 10^–3^ and 2.26 × 10^–3^ for random multiplexing and spatial multiplexing with TAP-GSA, respectively. Furthermore, the exposure times needed in the image sensor with the same dynamic range for recording the hologram of a pinhole for random multiplexing and spatial multiplexing with TAP-GSA are 71 and 42 ms*,* respectively, which is an improvement of ~ 1.7 times.

**Figure 8:**
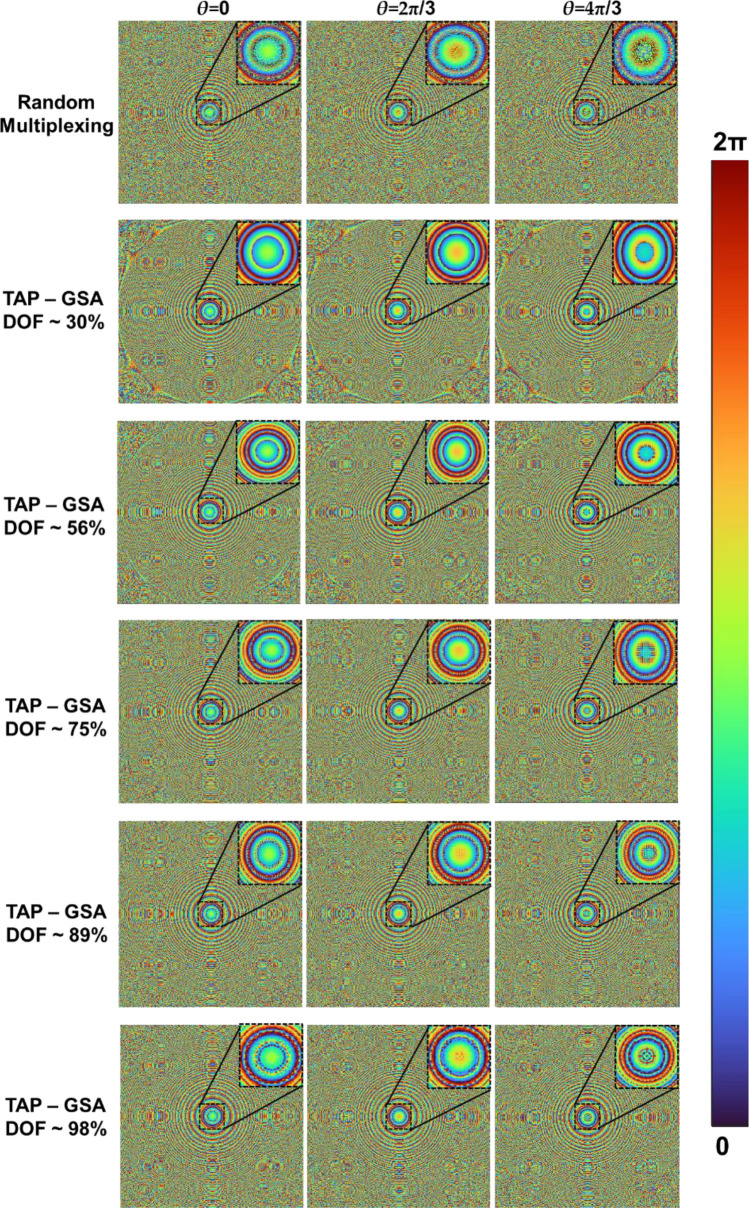
Images of the phase masks synthesized for DOF ~ 30, 56, 75, 89 and 98% with phase shifts *θ* = 0, 2π/3 and 4π/3. Two diffractive lenses with focal lengths of 11 and 46 cm are multiplexed.

**Figure 9:**
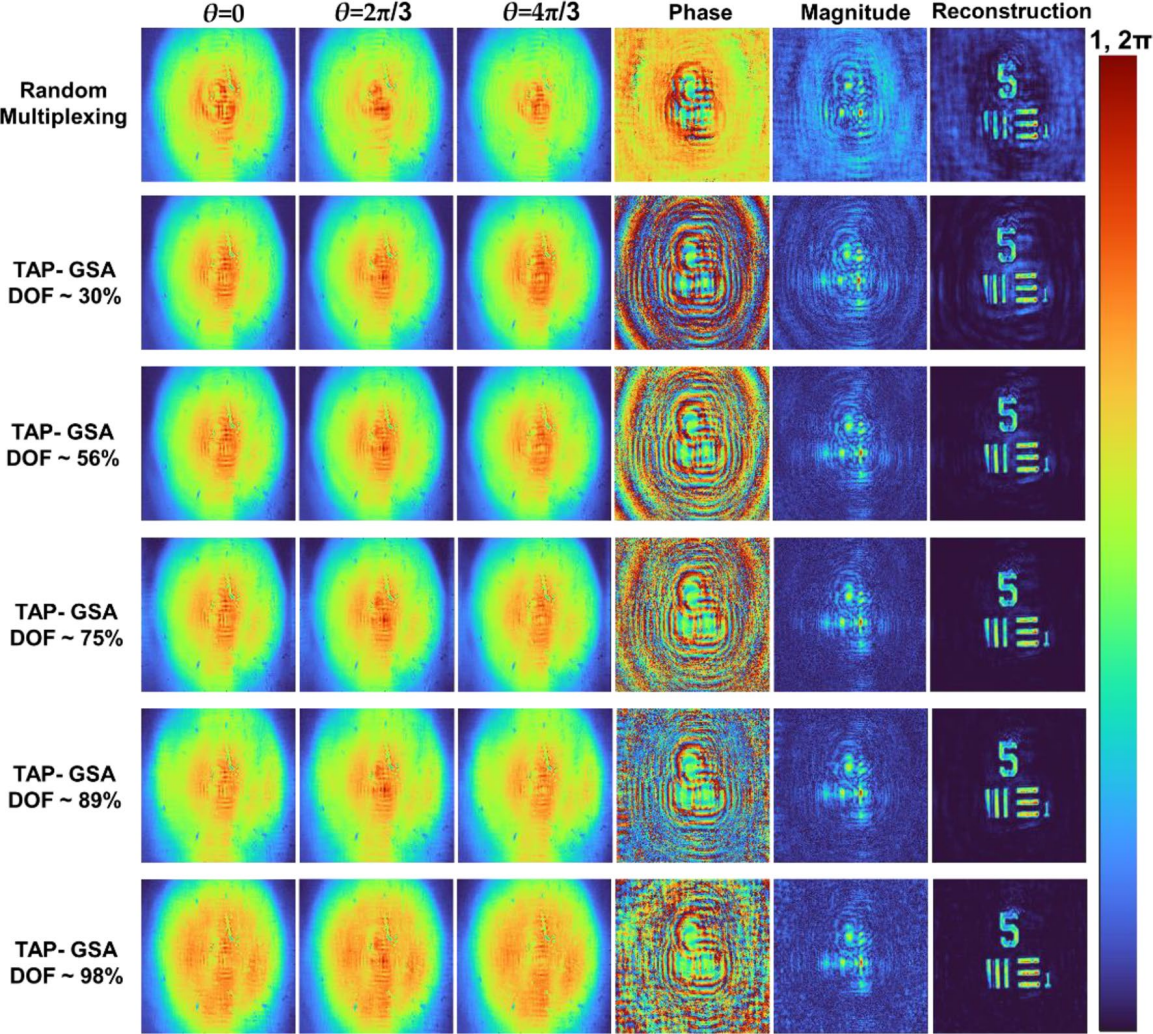
Images of the phase-shifted holograms *θ* = 0, 2π/3 and 4π/3 recoded with the masks of Fig. 8, magnitude and phase of the complex holograms and reconstruction results with DOF values of ~ 30, 56, 75, 89 and 98%.

The recording configuration was modified by changing the focal length of the two lenses to 11 and 46 cm for the next experiment. In this configuration, the USAF object was mounted in place of the pinhole object. This time, the DOF was varied in the TAP-GSA to the following values ~ 30, 56, 75, 89 and 98%, and the mask was synthesized after 100 iterations. The images of the phase masks for the above values of DOF from TAP-GSA are shown in Fig. 8. As shown in Fig. 8, the phase mask similarity to the ideal phase function decreases as the DOF increases, as expected. The optical experiment was repeated using phase masks designed using different DOFs. The images of the phase-shifted holograms, the magnitude and phase of the complex holograms and the reconstruction results for the different DOF values are shown in Fig. 9. The reconstruction distance was approximately 30 cm. The ABN values for the random multiplexing method and spatial multiplexing using TAP-GSA with DOF values of ~ 30, 56, 75, 89 and 98% are calculated as 22.9 × 10^–3^, 4.8 × 10^–3^, 2.3 × 10^–3^, 1.7 × 10^–3^, 4.0 × 10^–3^, and 5.3 × 10^–3^, respectively. The exposure times for recording the hologram of the USAF object for random multiplexing and spatial multiplexing with TAP-GSA were 72 and 48 ms*,* respectively. When the DOF was varied, there was mild to no change in the exposure time.

**Figure 10:**
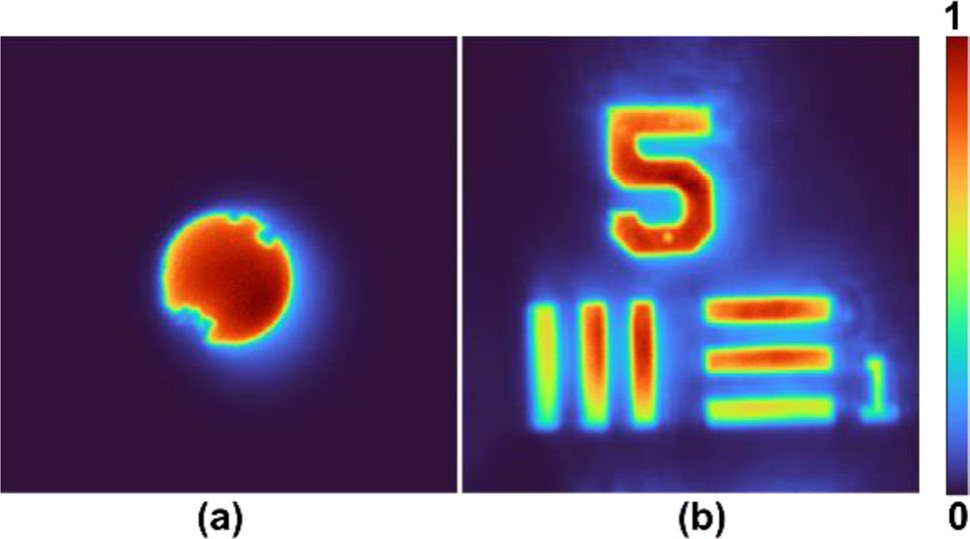
Direct imaging result of (a) pinhole and (b) USAF object.

Comparing the reconstruction results of pinhole and USAF objects for random multiplexing and spatial multiplexing with TAP-GSA shows a significant improvement in SNR with the proposed method. Since such a difference in noise levels between random multiplexing and spatial multiplexing with TAP-GSA for a small object such as a pinhole was observed, the noise difference is expected to increase for complicated objects. While the ABN increased for random multiplexing, it was at the same level for spatial multiplexing with TAP-GSA. The direct imaging results for the pinhole and USAF objects are shown in Fig. 10a and b, respectively. The ABNs for the pinhole and USAF objects were 0.15 × 10^–3^ and 0.53 × 10^–3^, respectively.

### FINCH with maximum optical path difference

**Figure 11:**
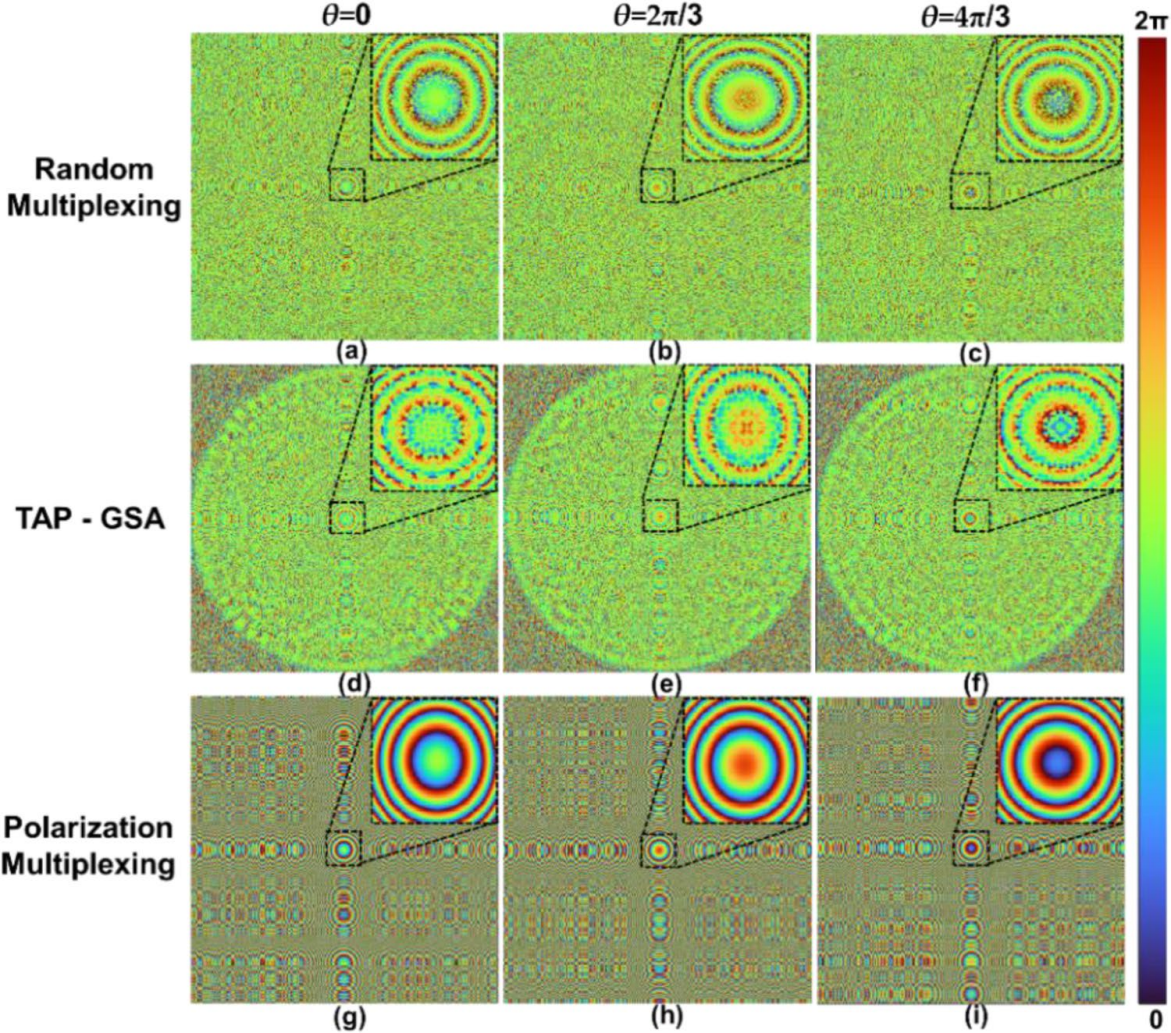
Phase masks for the setup of the maximum path difference. The upper line shows masks designed using the random multiplexing method with phase shifts (**a**) *θ* = 0, (**b**) 2π/3 and (**c**) 4π/3. Middle line: phase masks designed using the TAP-GSA method with phase shifts of (**d**) *θ* = 0, (**e**) 2π/3 and (**f**) 4π/3. Lower line: phase masks for the polarization multiplexing method for (**g**) *θ* = 0, (**h**) 2π/3 and (**i**) 4π/3.

In this configuration, the focal lengths of the diffractive lenses were 8.9 cm and infinity. The effective diameter of the SLM used is therefore approximately 7 mm. The two lenses were combined using random multiplexing as well as spatial multiplexing with TAP-GSA. For polarization multiplexing, a single diffractive lens with a focal length of 8.9 cm was displayed on the SLM. The images of the phase masks for random multiplexing, spatial multiplexing with TAP-GSA (DOF ~ 10%) and polarization multiplexing are shown in Fig. 11. As polarizer P1 was oriented at 45° with respect to the active axis of the SLM, only approximately 50% of the light was focused by the diffractive lens, while the remaining part of the incoming light was not modulated. A second polarizer mounted before the image sensor with orientation at 45° with respect to the active axis of the SLM ensures interference between the light focused by the diffractive lens and the unmodulated part. Once again, two objects, namely, the pinhole and USAF object, were used as test objects. The exposure times needed for recording the hologram of the pinhole object with a full dynamic range for random multiplexing, spatial multiplexing with TAP-GSA and polarization multiplexing were 615, 515 and 983 ms, respectively. The exposure times needed for recording the hologram of the USAF object with full dynamic range for random multiplexing, spatial multiplexing with TAP-GSA and polarization multiplexing were 440, 384 and 861 ms, respectively.

**Figure 12:**
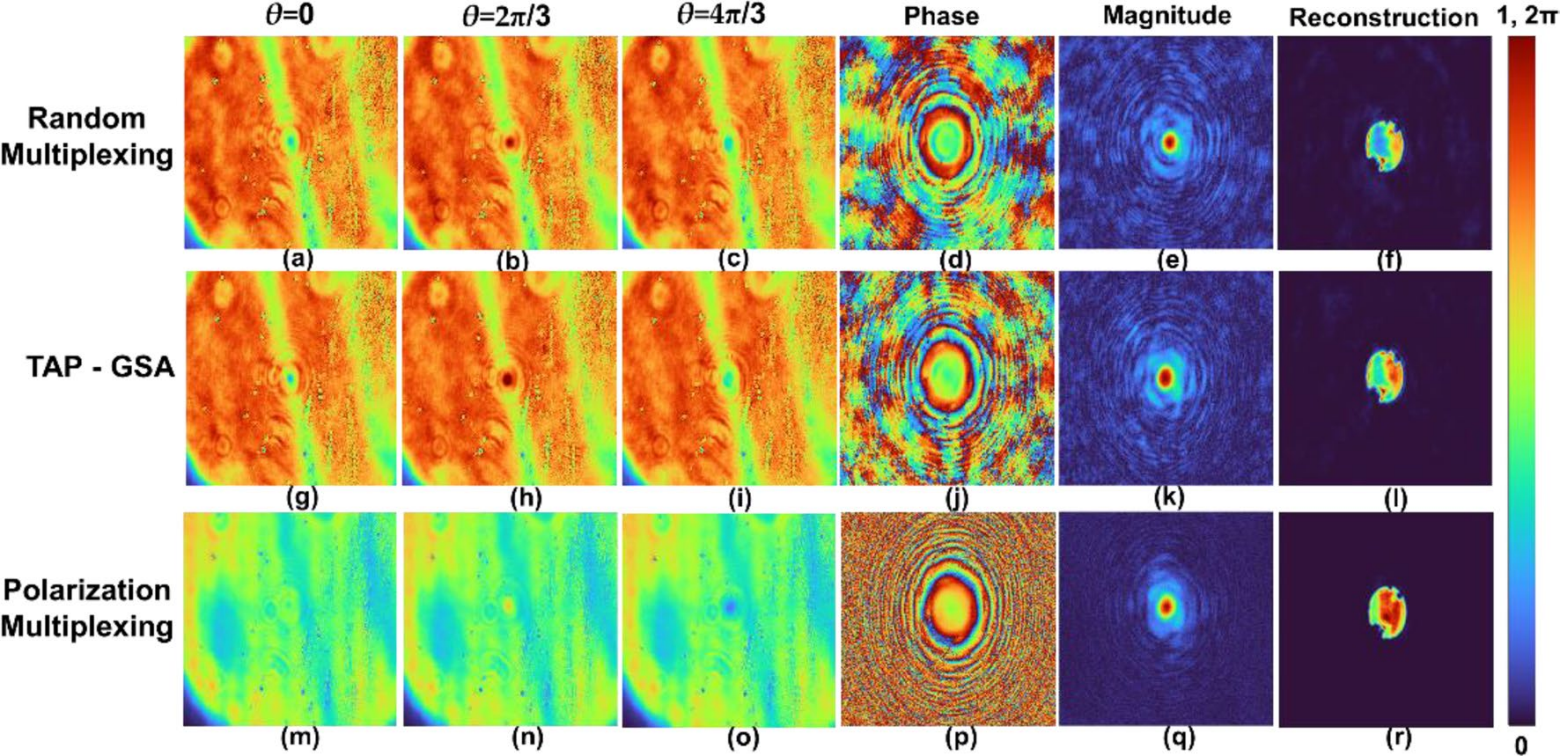
Holograms of the pinhole with maximum path difference. The upper line shows holograms of the random multiplexing method with phase shifts of (**a**) *θ* = 0, (**b**) 2π/3 and (**c**) 4π/3, (**d**) phase, (**e**) magnitude, of the complex hologram and (**f**) reconstructed image of the pinhole. Holograms of the TAP- GSA method with phase shifts of (**g**) *θ* = 0, (**h**) 2π/3 and (**i**) 4π/3, (**j**) phase, (**k**) magnitude, of the complex hologram, and (**l**) reconstructed image of the pinhole. Holograms of the polarization multiplexing method with phase shifts of (**m**) *θ* = 0, (**n**) 2π/3 and (**o**) 4π/3, (**p**) phase, (**q**) magnitude, of the complex hologram, and (**r**) reconstructed image of the pinhole.

**Figure 13:**
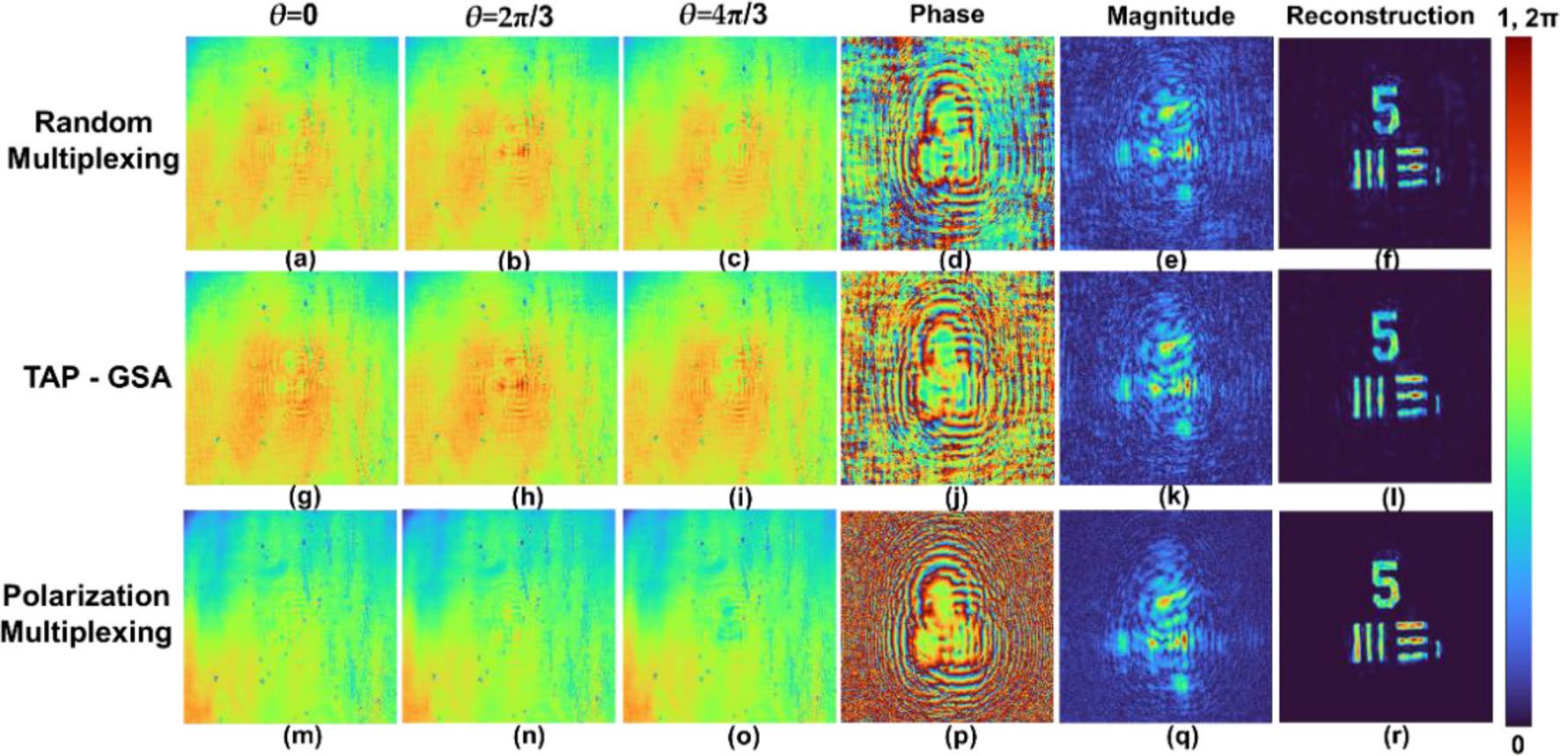
Holograms of the USAF target with maximum path difference. The upper line shows holograms of the random multiplexing method with phase shifts of (**a**) *θ* = 0, (**b**) 2π/3 and (**c**) 4π/3, (**d**) phase, (**e**) magnitude, of the complex hologram and (**f**) reconstructed image of the USAF target. Holograms of the TAP- GSA method with phase shifts of (**g**) *θ* = 0, (**h**) 2π/3 and (**i**) 4π/3, (**j**) phase, (**k**) magnitude, of complex hologram, and (**l**) reconstructed image of the pinhole. Holograms of the polarization multiplexing method with phase shifts of (**m**) *θ* = 0, (**n**) 2π/3 and (**o**) 4π/3, (**p**) phase, (**q**) magnitude, of the complex hologram, and (**r**) reconstructed image of the USAF object.

The images of the phase-shifted holograms *θ* = 0, 2π/3 and 4π/3 for the pinhole object are shown in Fig. 12a–c for the random multiplexing method. The magnitude and phase of the complex hologram and the reconstruction result by Fresnel back propagation are shown in Fig. 12d–f, respectively. A similar set of images for spatial multiplexing with TAP-GSA is shown in Fig. 12g–l. The same set of images for polarization multiplexing is shown in Fig. 12m–r. The images of the phase-shifted holograms *θ* = 0, 2π/3 and 4π/3 for the USAF object are shown in Fig. 13a–c for the random multiplexing method. The magnitude and phase of the complex hologram and the reconstruction result by Fresnel back propagation are shown in Fig. 13d–f, respectively. Similar images for spatial multiplexing with the TAP-GSA are shown in Fig. 13g–l. A similar set of images for polarization multiplexing is shown in Fig. 13m–r.

**Figure 14:**
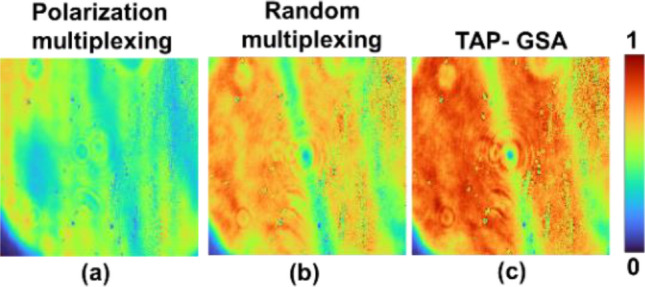
Holograms of a pinhole with an exposure time of 1587.4 ms recorded for the (**a**) polarization multiplexing method, (**b**) random multiplexing method, and (**b**) spatial multiplexing with (**c**) TAP-GSA method.

For all three multiplexing cases, the reconstruction distance was approximately 9 cm. The ABN was measured for all three cases for the pinhole and USAF objects. For pinholes, the ABN of random multiplexing, spatial multiplexing by TAP-GSA and polarization multiplexing are 3.27 × 10^–3^, 2.32 × 10^–3^ and 0.41 × 10^–3^, respectively. For the USAF object, the ABN of random multiplexing, spatial multiplexing by TAP-GSA and polarization multiplexing are 2.91 × 10^–3^, 2.37 × 10^–3^ and 0.62 × 10^–3^, respectively. The SNR is the highest for the polarization multiplexing method, while TAP-GSA is better than random multiplexing. However, the power requirement is the lowest for TAP-GSA compared to both random multiplexing and polarization multiplexing. The holograms of the pinhole with the same exposure time of 1587.4 ms were recorded for polarization multiplexing, random multiplexing and spatial multiplexing with TAP-GSA, as shown in Fig. 14. The power requirements of polarization multiplexing and random multiplexing are higher than those of the proposed method. The values of the exposure time (256 levels) and ABN for FINCH with reduced path difference and maximum path difference and direct imaging for pinhole and USAF objects are given in Tables 1 and 2.

**Table 1:** Exposure time and ABN for FINCH with maximum and reduced path difference and direct imaging (DI) for random multiplexing (RM), TAP-GSA and polarization multiplexing (PM) for pinhole object.

Properties	FINCH reduced path difference (Pinhole)		FINCH maximum path difference (Pinhole)			DI (Pinhole)
	RM	TAP-GSA	RM	TAP-GSA	PM	
Exposure time (ms)	71	42	615	515	983	2
ABN (× 10^–3^)	2.36	2.26	3.27	2.32	0.41	0.15

**Table 2:** Exposure time and ABN for FINCH with maximum and reduced path difference and direct imaging (DI) for random multiplexing (RM), TAP-GSA and polarization multiplexing (PM) for USAF object.

Properties	FINCH reduced path difference (USAF object)						FINCH maximum path difference (USAF object)			DI (USAF Object)
	RM	TAP-GSA (DOF 30%)	TAP-GSA (DOF 56%)	TAP-GSA (DOF 75%)	TAP-GSA (DOF 89%)	TAP-GSA (DOF 98%)	RM	TAP-GSA	PM	
Exposure time (ms)	72	48	48	48	48	48	440	381	861	3
ABN (× 10^–3^)	22.9	4.8	2.3	1.7	4	5.3	2.91	2.37	0.62	0.53

### Super-resolution

**Figure 15:**
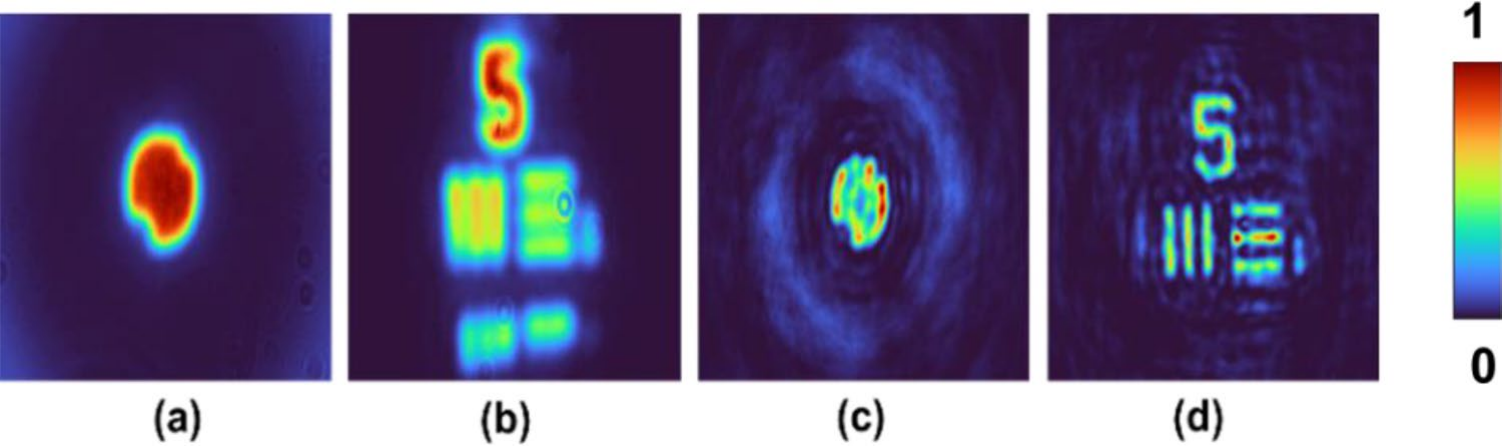
Direct imaging results of the (**a**) pinhole and (**b**) USAF object. FINCH reconstruction results for the spatial multiplexing method based on TAP-GSA of the (**c**) pinhole and (**d**) USAF objects.

One of the main advantages of FINCH in comparison to direct imaging systems is the capability to image objects with an improved resolution. To verify if this capability is retained in the spatial multiplexing method with TAP-GSA, the diameter of the diffractive lens displayed on the SLM was kept at ~ 1.5 mm such that the two test objects are not resolved in direct imaging mode. The images of the pinhole and USAF object are shown in Fig. 15a and b, respectively. The same diameter constraint was applied to the diffractive elements synthesized using TAP-GSA. The reconstructed images of the two objects for FINCH with maximum path difference are shown in Fig. 15c and d. The enhanced resolution of TAP-GSA can be clearly seen in the case of FINCH, indicating that the proposed method retains the improved resolution capability of FINCH.

## Conclusion and future perspectives

A new computational algorithm called TAP-GSA has been developed to design multiplexed diffractive lenses for FINCH. The TAP-GSA was executed with 100 iterations in most cases, and the time of execution for a matrix size of 1200 × 1200 pixels was ~ 35 s in a computer with 32 GB RAM, a 64-bit operating system and an 11th Gen Intel(R) Core (TM) i7-11700 @ 2.50 GHz processor. The proposed method was evaluated in simulation and optical experiments in two main optical configurations of FINCH. The new method with TAP-GSA was found to exhibit lower reconstruction noise than the widely used random multiplexing method. Compared to the polarization multiplexing method of FINCH, the proposed method has higher reconstruction noise. However, the optical power efficiency of the spatial multiplexing method based on TAP-GSA is better than that of both random multiplexing and polarization multiplexing. As the noise level for the new method with TAP-GSA is better than random multiplexing and the power requirements are lower, the new method will enable implementing FINCH for experiments involving high temporal resolution. The polarization multiplexing method was not implemented in the first configuration of FINCH with a reduced path difference, as either an additional refractive lens is needed or the location of the refractive lens (element 8 in Fig. 5) needs to be shifted to achieve the optimal beam overlap condition. The above change may influence the SNR and the light throughput. The special resolution capability of FINCH was evaluated with the proposed method, and a resolution enhancement was noticed.

The simulation results and the derived conclusions are true only for that particular optical configuration and simulation conditions. When the optical configuration is varied involving a change in distance, aperture size, wavelength, and pixel size, mild to significant variations in the results were observed with the optimal DOF varying between 0 and 100%. When the DOF is 100%, the pure phase mask becomes random-like, resulting in scattering of light, and on the other hand, when the DOF is small, the pure phase condition is not achieved, resulting in higher reconstruction noise. There is a trade-off between the above two effects that needs to be considered. Therefore, it is necessary to run the algorithm for the exact experimental conditions and optical configuration. The simulation results for the second configuration with varying DOF are given in the first part of the supplementary section. The noise level in the case of random multiplexing was observed to be higher than that in a previous report^23^.

The developed TAP-GSA will not only benefit FINCH but also be extended to other imaging techniques, such as I-COACH with Airy beams, dot patterns and multiplane direct imaging^25^. To understand the impact of this work on general diffractive optics design, binary versions of phase masks with random multiplexing and spatial multiplexing with TAP-GSA for FINCH with a reduced path difference configuration were fabricated using photolithography and tested. The details of the fabrication and testing results are given in the second part of the supplementary section. A proof-of-concept simulation study was carried out for multiplexing five diffractive lens functions using TAP-GSA, and the results agreed with the conclusions drawn from the results obtained for FINCH. The results are given in the supplementary materials. We believe that the current development will be valuable for future implementation of FINCH technology in both fluorescence microscopy and imaging applications^26^.

## Supplementary Information

Supplementary Information.

## Data Availability

Data underlying the results presented in this paper are not publicly available at this time but may be obtained from the corresponding author Vijayakumar Anand upon reasonable request.
