# Supplementary material for: Enhanced design of multiplexed coded masks for Fresnel incoherent correlation holography
Source: Sci Rep. 2023 May 6;13:7390. doi: 10.1038/s41598-023-34492-2 (PMC10164182; doi:10.1038/s41598-023-34492-2)
Supplement: Supplementary file 1 — Supplementary Information. [file 41598_2023_34492_MOESM1_ESM.pdf]

Supplementary document of "Enhanced Design of Multiplexed Coded Masks for Fresnel Incoherent Correlation Holography"

Shivasubramanian Gopinath,[1] Andrei Bleahu,[1] Tauno Kahro,[1] Aravind Simon John Francis Rajeswary,[1] Ravi Kumar,[2] Kaupo Kukli,[1] Aile Tamm,[1] Joseph Rosen,[1,3] and Vijayakumar Anand,[1,4,*]

[1]*Institute of Physics, University of Tartu, W. Ostwaldi 1, 50411 Tartu, Estonia*
[2]*Department of Physics, SRM University-AP, Amaravati 522502, Andhra Pradesh, India*
[3]*School of Electrical and Computer Engineering, Ben-Gurion University of the Negev, Beer-Sheva 8410501, Israel*
[4]*Optical Sciences Center and ARC Training Centre in Surface Engineering for Advanced Materials (SEAM), School of Science, Computing and Engineering Technologies, Optical Sciences Center, Swinburne University of Technology, Hawthorn, Melbourne, VIC 3122, Australia*
**vijayakumar.anand@ut.ee*

1. Simulation results

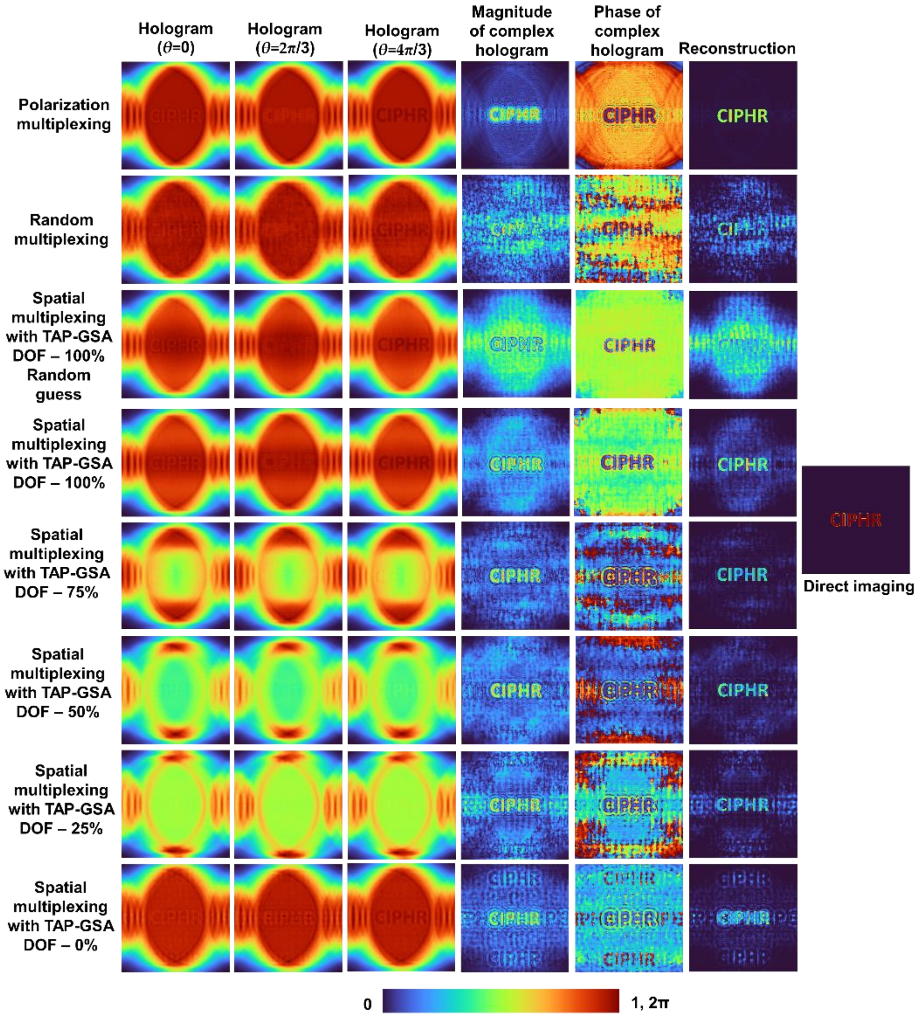

Figure S1 Simulation results of random multiplexing, polarization multiplexing and spatial multiplexing using TAP-GSA with different DOFs of 100%, 75%, 50%, 25% and 0% with the ideal initial guessed phase matrix and DOF 100% with the random guessed phase matrix.

The simulation was repeated for the simplified version of the first configuration shown in Fig. 2(a) for polarization multiplexing, random multiplexing and spatial multiplexing with DOFs of 0%, 25%, 50%, 75% and 100% with the ideal initial guessed phase matrix and random initial guess phase matrix. In this simulation, a matrix consisting of 1200×1200 pixels was used with a pixel size of 8 μm and a wavelength λ = 632.8 nm. The first configuration was simulated for $z_1 = f_1 = 1000$ cm, $z_2 = 17.8$ cm, and $f_2 = 8.9$ cm. Except for the case with the initial guess as a random matrix, all other cases demonstrated an SNR higher than the random multiplexing method. This behavior is different from the results shown in Figure 3.

2. Fabrication and testing of phase masks

The phase masks designed for FINCH with reduced path difference ($f_1 = 14$ cm and $f_2 = 25$ cm) were binarized with a pixel size of 8 μm and a length of 9.6 mm and converted from a bitmap file to Graphic Design System (GDSII) using the trial version of LinkCAD software [S1]. The images of the binary masks for random multiplexing and spatial multiplexing with TAP-GSA are shown in Figs. S2(a) and S2(b), respectively. The computer-aided design (CAD) of a section of the randomly multiplexed device is shown in Fig. S2(c). The thickness t of the photoresist was optimized for a wavelength of $\lambda = 650$ nm as $t = \lambda/2(n\text{-}1)$, where n is the refractive index of the photoresist. The devices were fabricated using a photolithography technique, where the fabrication of masks was carried out in an ISO5 cleanroom by using a Maskless Aligner (Heidelberg Instruments, μMLA 100, Germany) with dose control of the light source at 390 nm. Positive photoresist (AR-P 3510T, Allresist, Germany) was spin coated (4000 rpm, 60 s) onto cleaned glass substrates and softly baked on a hot plate at 100° C for 60 s. To improve the adhesion of the photoresist to the glass substrates, the promoter AR 300-80 new (Allresist, Germany) was used, and AR 300-44 (Allresist, Germany) was used for developing the UV irradiated structures. The optical microscope images of the fabricated devices corresponding to Fig. S2(a) are shown in Figs. S3(a) and S3(c). The optical microscope images of the fabricated devices corresponding to Fig. S2(b) are shown in Figs. S3(b) and S3(d).

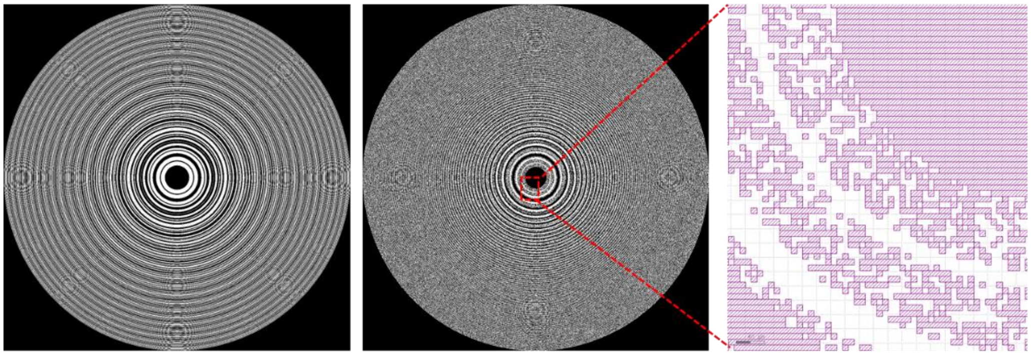

(a) (b) (c)

Figure S2. Binary phase mask for (a) spatial multiplexing with TAP-GSA and (b) random multiplexing for FINCH with reduced path difference. (c) CAD diagram of a section of the randomly multiplexed diffractive lens.

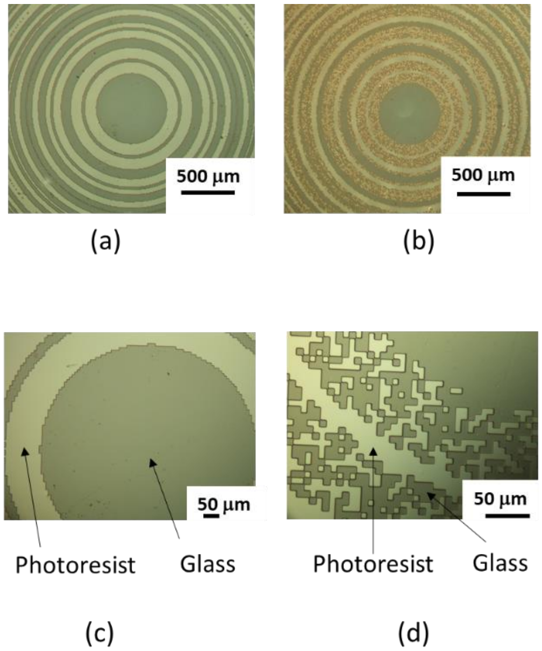

Figure S3. Optical microscope images of the central part of the binary phase mask for (a)(c) spatial multiplexing with TAP-GSA and (b)(d) random multiplexing for FINCH with reduced path difference.

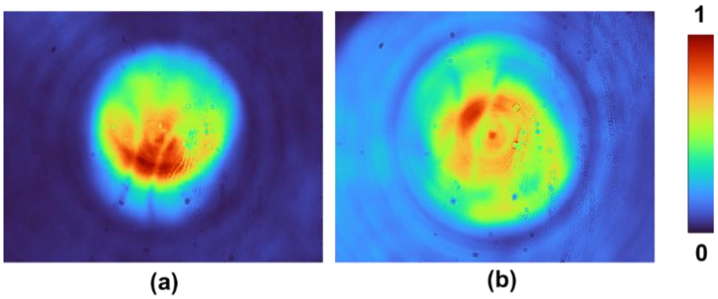

Figure S4. Holograms recorded for the pinhole with 100 μm using FINCH with reduced path difference with (a) spatial multiplexing with TAP GSA and (b) random multiplexing method.

The fabricated images were tested in the same optical setup shown in Figure 5. The beamsplitter before the SLM divides the beam into two. The fabricated devices were mounted perpendicular to the SLM, and the image sensor wave was moved to record the holograms. The hologram images for the 100 μm pinhole recorded using the image sensor are shown in Figures S4(a) for the TAP-GSA and S4(b) for the random multiplexing. From the experimental results, it is evident that spatial multiplexing with TAP-GSA has a better concentration of light than the randomly multiplexed case. Note that in this experiment, only a single camera shot hologram is created by one phase mask for each multiplexing method, and hence, a phase-shift-based reconstruction cannot be performed. Nevertheless, the similarity of Fig. S4(a) with TAP-GSA multiplexing to the holograms of a pinhole shown in Fig. 7(g)-7(i) is higher than the similarity of Fig. S4(b) with random multiplexing to the holograms of a pinhole shown in Fig. 7(a)-7(c).

3. Design of phase masks for the generation of five non-collinear beams with different wavefront curvatures

A simulation study was carried out with the following conditions: matrix size of 500×500 pixels, pixel size of 10 μm and wavelength $\lambda = 632.8$ nm. Five diffractive lens functions with focal distances of 15 cm, 14.35 cm, 13.64 cm, 12.86 cm and 12 cm to image objects located at 30 cm, 27.5 cm, 25 cm, 22.5 cm and 20 cm, respectively, from the lens and an image distance of 30 cm were designed. A unique linear phase is attached to each diffractive lens function to

prevent collinear propagation. The phase images of the five diffractive lens functions are shown in Figure S5(a)-(e). The amplitude and phase of the ideal complex mask obtained by summing the phase of the five diffractive lens functions are shown in Figure S5(f) and S5(g). The phase-only diffractive mask obtained by random multiplexing of the five diffractive lens functions is shown in Figure S5(h). The amplitude constraint for TAP-GSA at the sensor plane was obtained from the intensity distribution corresponding to the object plane at 25 cm. The phase-only diffractive elements obtained using TAP-GSA with DOF – 68% and 100% are shown in Figures S5(i) and S5(j), respectively. The simulated images of the intensity distributions at the sensor plane for object distances of 30 cm, 27.5 cm, 25 cm, 22.5 cm and 20 cm for the ideal case, random multiplexing and TAP-GSA are shown in Figure S6. The intensity distribution obtained for TAP-GSA with 68% DOF has less background noise than random multiplexing for all cases. The results for TAP-GSA with 100% DOF are poor except for the object distance of 25 cm, as the phase information was not included.

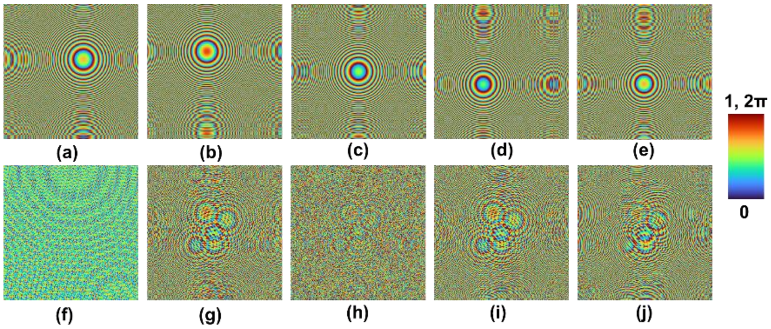

Figure S5 (a)-(e) Phase images of the five diffractive lens functions. (f) Amplitude and (g) phase of the ideal complex mask. Phase of the multifunctional diffractive mask obtained by (h) random multiplexing and TAP-GSA with (i) 68% DOF and (j) 100% DOF.

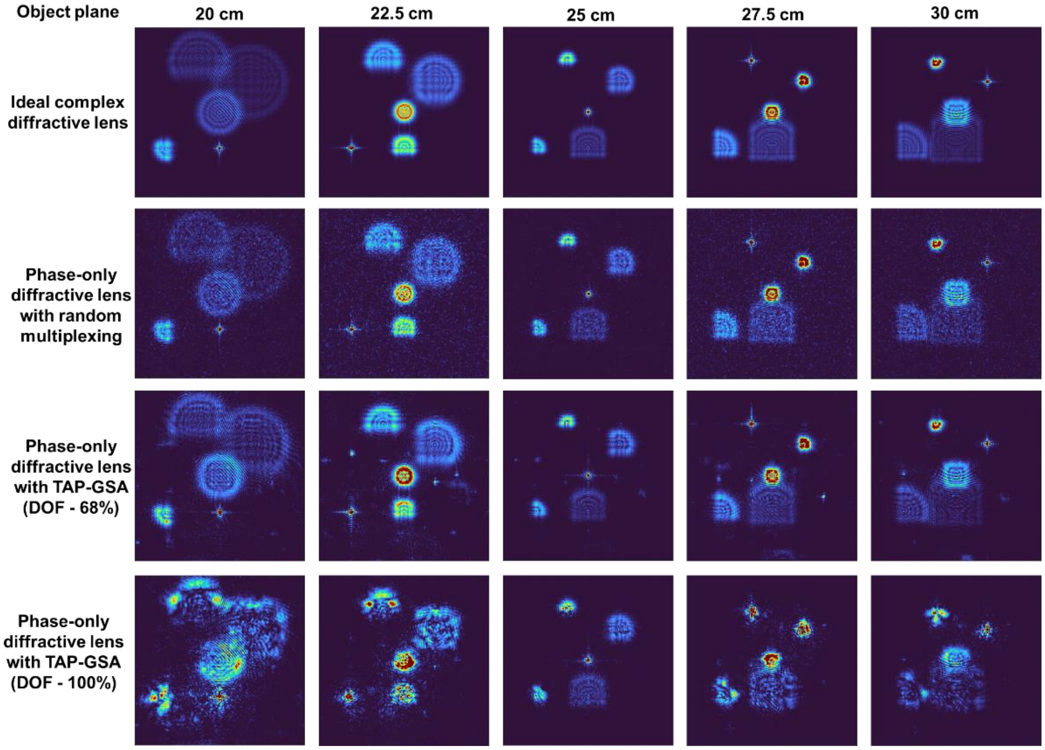

Figure S6 Simulation results of imaging at object planes located at 20 cm, 22.5 cm, 25 cm, 27.5 cm and 30 cm with a complex diffractive lens mask, phase-only diffractive mask with random multiplexing and TAP-GSA with 68% and 100% DOF.

References

[S1] A. Vijayakumar and S. Bhattacharya, Design and Fabrication of Diffractive Optical Elements with MATLAB (SPIE, 2017).
